# Cincinnati College of Dental Surgery

**Published:** 1845-03

**Authors:** 


*Cincinnati College of Dental Surgery.*—We have been informed that the legislature of Ohio, at its last session, chartered a Dental College, to be located in Cincinnati. With regard to the provisions of the charter or number of professorships, we are as yet ignorant. We, however, wish those engaged in this most commendable enterprise, every success, and so soon as we shall be made acquainted with the plan of instruction proposed to be adopted, and the names of the professors, we shall speak of it more at length. Until then, all we can do, is to announce the fact of the institution of the school. [*Balt. Ed.*

